# Nurse Clinical Reasoning Scale: Spanish Translation and Psychometric Properties

**DOI:** 10.1002/nop2.70196

**Published:** 2025-03-23

**Authors:** Ana Pérez‐Perdomo, Nuria Fabrellas, Adelaida Zabalegui

**Affiliations:** ^1^ Hospital Clinic of Barcelona Barcelona Spain; ^2^ Universitat de Barcelona (UB) Barcelona Spain

## Abstract

**Aim:**

To translate the Nurse Clinical Reasoning Scale into Spanish, examine their psychometric properties and to determine the clinical reasoning levels of Spanish nurses.

**Design:**

The original Nurse Clinical Reasoning Scale instrument was translated into Spanish, with the objective of analysing its contents, semantics, and technical and conceptual equivalence. A cross‐sectional multicentre study with an online questionnaire survey of registered nurses working in Spain was used to identify the levels of clinical reasoning among respondents.

**Methods:**

The original scale was translated from the English language into the Spanish language through six steps following the guidelines of Sousa and Rojjanasrirat. Data was collected from April 2022 to June 2023 through an anonymous online questionnaire.

**Results:**

Five competitive and public Spanish hospitals participated in this study. The sample included 153 registered nurses. The validity for the entire scale indicated an excellent result. The general level of clinical reasoning among nurses in this study was good. Cronbach's alpha of the Spanish version was practically the same as the original version.

**Patient or Public Contribution:**

The Nurse Clinical Reasoning Scale is a valid and reliable instrument, and the Spanish version can be used to evaluate the clinical reasoning levels of nurses in a Spanish context. The tool will help to identify areas for improvement in clinical reasoning in nurses and may improve their patient care.

## 1 Introduction

Improvements in healthcare make that life expectancy is increasingly longer, with more multimorbidity's (Dominguez et al. 2021). Nurses must face more complex clinical situations, and it is more difficult to have the appropriate skills for it. Patient safety can be affected when there is a poor clinical reasoning (CR) that results from incorrect decisions in patient care (Holder 2018). CR is a cognitive process that helps nurses in patient care, guiding them in problem‐solving and decision‐making (Hunter and Arthur 2016). CR represents a dynamic process that nurses employ to ensure sound clinical decisions and optimal outcomes. This process requires collecting data, cognitively processing the information, understanding a patient's problem or situation, planning and implementing evidence‐based interventions, evaluating results, reflecting and learning from the process (Levett‐Jones et al. 2010).

Patients' quality of care and the possibility of their improvement depend directly on the CR nursing skills. Therefore, it is necessary to have measuring clinical reasoning instruments (Bae et al. 2023). There are tools that evaluate clinical reasoning, but most of them evaluate clinical reasoning levels in other specialities such as medicine or physiotherapy. For instance, a recent review study shows that the most suitable clinical reasoning scales for the medical field are the script concordance test or the Lasater clinical judgement rubric (Brentnall et al. 2022). However, none of these published or registered instruments are adapted to Spanish nurses.

We searched clinical reasoning tools specific for nurses on four electronic databases: PUBMED, Web of science, Cinahl and Scielo, selecting ten instruments. To determine the most robust instruments, we analysed their psychometric properties [validity, internal consistency reliability (*α* de Cronbach) and test–retest stability]. Our review was also conducted considering their accuracy and usability in registered nurses.

Most of the instruments designed to measure CR in nursing have been described as rubrics or clinical examinations lacking a solid theoretical foundation (Kojabadi et al. 2023). We conducted a systematic review of available tools for assessing nurses' clinical reasoning abilities. Our search revealed several valid, reliable and robust instruments, including the Lasater Clinical Judgement Rubric (LCJR) (Lasater 2007), which evaluates clinical judgement based on Tanner's four phases; the Clinical Reasoning Evaluation Simulation Tool (CREST) (Liaw et al. 2018), designed to measure clinical reasoning skills in recognising and responding to clinical deterioration in simulated environments; or the Script Concordance Test (SCT) (Charlin et al. 2000), which assesses clinical reasoning by evaluating how healthcare professionals organise knowledge (Liou et al. 2016). After careful consideration, we selected the Nurse Clinical Reasoning Scale (NCRS) for translation into Spanish due to its strong theoretical underpinning (Liou et al. 2016). The NCRS is based on the clinical reasoning cycle model and entails eight crucial phases: look, collect, process, decide, plan, act, evaluate and reflect (Levett‐Jones et al. 2010) Though each phase is presented as a distinct and separate element, nurses often move back and forth between phases before acting and evaluating the outcomes. This dynamic approach aligns closely with the complex nature of clinical reasoning in real‐world nursing practice, making the NCRS a particularly valuable tool for assessing and developing this critical skill in nursing education and professional development.

The NCRS, developed in Taiwan in 2015, is a brief and straightforward questionnaire simple to comprehend and complete. The tool has been designed to evaluate nurses clinical reasoning competences in a variety of clinical settings (Liou et al. 2016). The original scale was in Chinese, but for dissemination purposes, the scale was translated into English by a standard translation back translation. The NCRS has gained international recognition and has been translated into multiple languages, including Italian (Notarnicola et al. 2023), Persian (Kojabadi et al. 2023), Dutch (Janssen 2021) and Korean (Joung and Han 2017). Notably, all translated versions have maintained the original items and reported similar robust psychometric properties, highlighting its potential for cross‐cultural application and global impact. Given its strong psychometric properties, the NCRS is well suited for our study, as it is likely to provide reliable results in assessing nurses' clinical reasoning levels in Spain.

Translating the Nurse Clinical Reasoning Scale to Spanish is an essential step in pursuing the availability of instruments to measure the main clinical outcomes in patient‐centred care. The process of CR is important for improving optimal care within the standards of quality and safety care delivery. By effectively translating the NCRS scale, healthcare providers will be better equipped to understand and evaluate clinical reasoning skills in nurses. Therefore, healthcare providers could measure and potentiate their CR and, therefore, improve patient care.

In Spain, nurses hold a licence based on a baccalaureate of four years university degree that meets the requirements set by European regulations, so their licence is valid across the European Union countries (Cabrera and Zabalegui 2020). Nurses in Spain are highly trained and well prepared at level six of the European Quality Education classification system (EFQM). They are highly skilled and knowledgeable, with a deep understanding of patient care and medical procedures; however, according to the OECD report of 2023, the number of nurses per 1000 inhabitants in Spain is 6.3 is much lower than the European average of 9.2. In comparison, countries like Finland and Norway have triple the ratio of nurses per 1000 inhabitants than in Spain, with 18.9 and 18.3, respectively. Even neighbouring countries like France exceed the average with 9.7 nurses per 1000 inhabitants (OECD 2023). In this context, nurse's decision‐making time in clinical practice is much limited. Evidence suggests that characteristics such as a low patient/nurse ratio, a high proportion of nurses with a bachelor‐level education, and a better work environment are associated with lower mortality and failure‐to‐rescue (OECD 2023; Aiken et al. 2014). Given this circumstance, the NCRS tool will help us to know the nurses' clinical reasoning level and skills and especially to identify areas for improvement in CR in nurses in Spain, so new graduate nurses may improve their patient care.

The aims of this study are to translate the Nurse Clinical Reasoning Scale into Spanish and examine its psychometric properties; besides, we aim to know the clinical reasoning level of the Spanish nurses.

## 2 The Study

### 2.1 Design

The original instrument NCRS was translated into Spanish, with the objective of analysing its content, semantic, technical and conceptual equivalence following the guidelines of Sousa and Rojjanasrirat (Sousa and Rojjanasrirat 2011). Besides, we analysed the psychometric properties (content validity, test–retest reliability and internal consistency) of the Spanish version following the COSMIN checklist (Consensus‐based Standards for the selection of health status Measurement Instruments) guideline (Mokkink et al. 2010). Finally, a cross‐sectional multicentre study with an online questionnaire survey on registered nurses working in Spain was used to identify their level of CR.

### 2.2 The Instrument

The Nurse Clinical Reasoning Scale (NCRS) is a comprehensive tool designed to assess clinical reasoning skills in nurses, consisting of 15 items that evaluate key aspects of the logical problem‐solving process, including data collection from patients, recognition of patient problems and evaluation of nursing interventions. Each item is scored on a 5‐point Likert scale, ranging from 1 (strongly disagree) to 5 (strongly agree), with total scores ranging from 15 to 75 points; higher scores indicate better clinical reasoning ability. The items were developed by translating higher‐order thinking steps into question statements, with 1–4 items corresponding to each step of the clinical reasoning process. The NCRS does not have predetermined cut‐off points but instead provides an evaluation range where lower scores (closer to 15) suggest less developed clinical reasoning skills, while higher scores (closer to 75) indicate more advanced abilities (Liou et al. 2016). This continuous scoring method allows for flexibility in interpretation, enabling the assessment of individual progress over time or comparisons between groups, although it may complicate the establishment of absolute benchmarks of competence.

### 2.3 Method

#### 2.3.1 Translation Process

First, we obtained permission from the instrument author Liou et al. (2016) to use the scale in this study. The translation process used was based on the Sousa and Rojjanasrirat guideline (Sousa and Rojjanasrirat 2011). The translation team consists of two bilingual interpreters, two English bilingual philologists, two nurse educators and two clinical nurses. The original scale was translated from the original English language to the target Spanish language in six‐step process:

Step 1: Translation from English to Spanish.

The instrument was translated from English to Spanish, by two independent bilingual’ translators, with similar characteristics. The two independent translators fluent in both languages (bilinguals) and with knowledge and experience of the culture in both languages, produced two forward versions of the scale in Spanish.

Step 2: Synthesis of the two versions.

Both translations were examined by a third translator, after discussing with the first two translators and resolving discrepancies, the third translator synthesises the two translated versions into one. In this stage, the research team with the principal investigator collaborated in the process to resolve the following minor discrepancies:

Item 1: ‘*recoger*’ [pick up] versus ‘*recabar*’ [gather], the final decision was to include ‘*recoger*’ [pick up].

Item 5: ‘señales *o síntomas*’ [marks or symtoms] versus ‘*signos o sintomas*’ [signs or symtoms], the final decision was to include ‘*signos o sintomas*’ [signs or symtoms].

Item 13: ‘*mandadas por el médico’* [ordered by the doctor] versus *‘solicitadas por el médico*’ [requested by the doctor], the final decision was to include*'solicitadas por el médico’* [requested by the doctor].

Item 15: ‘conozco los pasos’ [I recognised the steps] versus ‘sé los pasos’ [I know the steps], the final decision was to include *‘conozco los pasos’* [I know the steps].

Step 3: Back translation.

Once the document was agreed in Spanish, two new independent bilinguals translators and two philologists of the English language produced two translations to English, completely blind to the original document.

Step 4: Differences between the two back translations.

The result of step three was discussed with the two translators and the research team. The content equivalence, structure and semantics were analysed. There were some cultural meaning differences in the structure of items eleven and twelve that were resolved by reaching the consensus of the translation team. After that, we compared the final document with the original document (both in English), and no significant differences between them were identified.

#### 2.3.2 Psychometric Analysis of the NCRS Spanish Version

The psychometric stages have been reviewed and structured based on the COSMIN checklist (Consensus‐based Standards for the selection of health status Measurement Instruments) (Mokkink et al. 2010). Following this guideline, the following psychometric properties have been evaluated in the study: content validity (including face validity), construct validity, cross‐cultural validity, internal consistency and stability of the instrument.

##### 2.3.2.1 Content Validity

- *Participants*: Fifteen participants were recruited to assess the instrument's face validity, content validity and semantic equivalence. The suggestions for changes from the participants were considered, and all modifications were accepted by the expert panel, ensuring that the words were equivalent without losing the original meaning of the sentences and items. The inclusion criteria required participants to be general ward nurses who are Spanish speakers with more than two years of experience in their field. Nurses working in emergency departments, surgical areas or intensive care units were excluded to ensure that participants had relevant experience with the specific context of the instrument being evaluated.
- *Face validity* was assessed qualitatively by asking participants to evaluate each item for clarity and ease of understanding. Participants were encouraged to provide feedback on whether the items appeared relevant and appropriate for measuring the intended constructs. This qualitative assessment allowed researchers to gather insights into how well the items resonated with the target population.
- *Item Analysis*: Participants evaluated each item for clarity and ease of understanding, providing suggestions for revisions as necessary. The following items were identified as unclear:
  ○ Item 2: 20% unclear
  ○ Item 4: 6.6% unclear
  ○ Item 6: 13.3% unclear
  ○ Item 8: 20% unclear
- *Suggested Revisions*:
  ○ Item 2: Change ‘Aplicar’ [apply] to ‘realizar’ [carry out]
  ○ Item 4: Change ‘anormal’ [abnormal] to ‘anómala’ [anomalous]
  ○ Item 6: Change ‘Mecanismo y desarrollo’ [mechanism and development] to ‘la etiología’ [aetiology]
  ○ Item 8: Change ‘Subyace’ [underlay] to ‘hay’ [there is]; change ‘el mecanismo’ [mechanism] to ‘la causa’ [the cause]
  ○ Item 12: Change ‘doctor’ [doctor] to ‘médico’ [physician]
- *Expert Panel Evaluation*

The research team reviewed the unclear items and participant suggestions. An expert panel was composed of two leading researchers in Spain, two pre‐doctoral researchers and eight registered nurses from hospitals. All proposed changes were accepted as they maintained the original meaning of the items. Each item was evaluated on a scale from 1 (not relevant) to 4 (very relevant). The CVI for each scale item was calculated based on the number of experts rating each item as a 3 or 4 (Sousa and Rojjanasrirat 2011). This process ensured that the items were not only relevant but also comprehensible to the intended audience.

- *Second Pilot Test*

The final version of the pilot test assessed the comprehensibility and clarity of the Spanish NCRS instrument. Participants were the same as in the previous step, and results indicated that none of the items was unclear, confirming readiness for psychometric testing.

##### 2.3.2.2 Reliability

- *Internal Consistency*: Reliability was analysed using Cronbach's alpha. The Cronbach's alpha measures the homogeneity and coherence of the survey statements. A Cronbach's alpha > 0.7 indicates an adequate internal consistency (Taber 2018).
- *Test–Retest Reliability*: A two‐week test–retest reliability analysis was conducted using the intraclass correlation coefficient (ICC) method. ICC is an indicator of test–retest items reliability. ICC < 0.9 means excellent reliability, between 0.75 and 0.9 means a good reliability, between 0.5 and 0.75 means a moderate reliability and less than 0.5 means poor reliability (Koo and Li 2016).

##### 2.3.2.3 Construct Validity

The construct validity of the questionnaire was verified through both exploratory factor analysis (EFA) and confirmatory factor analysis (CFA). The Kaiser‐Meyer‐Olkin statistic and Bartlett's test of sphericity were calculated to determine construct validity. The Kaiser‐Meyer‐Olkin measure of sampling adequacy and Bartlett's test of sphericity were used to determine the factor ability of the sample and the fit of the factor analysis. A Kaiser‐Meyer‐Olkin statistic value higher than 0.5 is acceptable (Taber 2018).

#### 2.3.3 Participants

The study sample included registered nurses from five different high‐technology public university Spanish hospitals.

Participation was voluntary via e‐mail. We sent the scale through the Google survey platform by the professional e‐mail net. The data were collected in an Excel database. The inclusion criteria for the participants were as follows: Spanish speaker registered nurses. On the other hand, the exclusion criteria were any people with professions different from nursing and nursing students.

To calculate the adequate sample size, we follow the Sousa and Rojjanasrirat (Sousa and Rojjanasrirat 2011) recommendation for general psychometric analysis. A sample of ten subjects per each instrument item was needed; therefore, as the NCRS instrument has 15 items, a minimum sample of 150 nurses was needed. Knowing that the response rate could be 50%, initially, 500 registered nurses were invited to participate, and finally, 153 nurses decided to participate (30.6%). They were invited to respond to a survey two times, at baseline and with an interval of three weeks to analyse the stability of the instrument. However, only 95 nurses answered the second‐time survey.

#### 2.3.4 Data Analysis

Data were collected from April 2022 to June 2023 through a cross‐sectional multicentred study, with an anonymous online questionnaire survey. The questionnaire included demographic characteristics of the sample: gender, age group, academic degree, working unit and name of institution. We measured the relationship between the demographic variables and the clinical reasoning to 75 points as the maximum punctuation. Data were analysed using the programme The Jamovi project (Jamovi n.d.) [Computer Software], retrieved from https://www.jamovi.org, and with the Microsoft Excel programme. Descriptive statistics analysis was carried out to analyse the demographic participant's characteristics.

#### 2.3.5 Ethical Consideration

The study has the approval of the Ethics and Research Committee (register number: HCB/2022/0955).

Before filling out the survey, all the participants were informed about the purpose of the study and the confidentiality of their participation with the following written statement:

Participation is voluntary and anonymous. The results of the survey will be published in a peer‐reviewed scientific journal. I have received information about the study. I have had time to consider my participation and to request more information. I have not been pressured to participate. I know that my participation is anonymous and voluntary and that some people will be able to see and process my data. I consent to my data being viewed and processed for this study.

## 3 Results of the Psychometric Analysis

### 3.1 Demographic Characteristics of the Participants

Five competitive, general and public Spanish hospitals participated in this study. The sample included 153 registered nurses, most of them females (86.9%). The 32.7% of the participants were between 50 and 59 years of age, and the rest of the participants had a mean age of 42.73 years. 61.4% of the sample had a master's degree. Most of the nurses worked in the surgical ward 41.7%. 109 of the study subjects had participated in the clinical training of nursing students. The demographic characteristics of the sample are described in Table 1.

**TABLE 1:** Demographic characteristics of the study sample.

Variable	*N*	%
*Gender*		
Female	133	86.9
Male	19	12.4
No gender	1	0.7
*Age group*		
20–29	29	19.0
30–39	28	18.3
40–49	41	26.8
50–59	50	32.7
60–65	5	3.3
*Academic degree*		
Only nursing bachelor's degree	33	21.6
Post‐graduate continuous education	14	9.2
Master's degree	94	61.4
PhD degree	12	7.8
*Ward*		
Medical and surgical ward	63	41.2
Others (psychiatry, haemodialysis operating room)	48	31.4
Obstetrics or paediatrics	12	7.8
Emergency or intensive care units	30	19.6

### 3.2 Content Validity

The CVI for this scale was rated at 0.875, while the overall content validity for the entire scale was 0.93, indicating excellent results (Salokivi et al. 2023).

### 3.3 Reliability

- *Internal Consistency*: Reliability was analysed using Cronbach's alpha, yielding a value of 0.93 for the Spanish version, comparable to the original scale, confirming that the adaptation reliably measures clinical reasoning in nurses (Taber 2018).
- *Test–Retest Reliability*: A two‐week test–retest reliability analysis was conducted using the intraclass correlation coefficient (ICC) method. A sample of 95 registered nurses was included in the analysis, and the results indicated that most of the sample was female, 86.3%, between 20 and 29 years old with a master's degree, and 46.3% worked in a medical and surgical ward. The demographic characteristics of the participants are represented in Table 2. The ICC result was found to be 0.523 (*p* < 0.001), indicating moderate internal stability (Koo and Li 2016).

**TABLE 2:** Demographic characteristics of the test–retest participants (*n* = 95).

Variable	*N*	%
*Age group*		
20–29	27	28.4
30–39	18	18.9
40–49	25	26.3
50–59	23	24.2
60–65	2	2.1
*Gender*		
Female	82	86.3
Male	11	11.6
No gender	1	1.1
Not say	1	1.1
*Academic degree*		
Only nursing degree	23	24.2
Post‐graduate	5	5.3
Master	59	62.1
PhD	8	8.4
*Ward*		
Medical and surgical ward	44	46.3
Others (psychiatry, haemodialysis operating room)	23	24.2
Obstetrics or paediatrics	3	3.2
Emergencies or intensive care	25	26.3

### 3.4 Construct Validity

The construct validity of the questionnaire was verified through both exploratory factor analysis (EFA) (Table 3) and confirmatory factor analysis (CFA) (Table 4).

**TABLE 3:** Exploratory factor analysis (EFA).

	Factor		Uniqueness
	1	2	
Item 1	0.502	0.330	0.639
Item 2	0.609	0.380	0.484
Item 3	0.826		0.298
Item 4	0.469		0.734
Item 5	0.547	0.475	0.476
Item 6	0.338	0.759	0.310
Item 7		0.784	0.324
Item 8	0.334	0.708	0.387
Item 9	0.625	0.387	0.460
Item 10	0.696	0.365	0.383
Item 11	0.544	0.508	0.446
Item 12	0.629	0.458	0.394
Item 13	0.358	0.641	0.462
Item 14	0.648	0.479	0.351
Item 15	0.369	0.682	0.398

*Note:* The ‘Minimum Residual’ extraction method was used in combination with a ‘varimax’ rotation.

**TABLE 4:** Confirmatory factor analysis (CFA).

Factor	Indicator	Estimator	EE	95% Confidence interval		*Z*	*p*
				Lower	Upper		
Factor 1	Item 1	0.548	0.0685	0.413	0.682	8.00	< 0.001
	Item 2	0.531	0.0543	0.424	0.637	9.77	< 0.001
	Item 3	0.458	0.0495	0.361	0.555	9.25	< 0.001
	Item 4	0.447	0.0713	0.308	0.587	6.28	< 0.001
	Item 5	0.535	0.0518	0.433	0.636	10.33	< 0.001
	Item 6	0.607	0.0552	0.499	0.715	10.99	< 0.001
	Item 7	0.599	0.0597	0.482	0.716	10.02	< 0.001
	Item 8	0.600	0.0583	0.485	0.714	10.28	< 0.001
	Item 9	0.528	0.0516	0.427	0.629	10.23	< 0.001
	Item 10	0.528	0.0488	0.432	0.623	10.82	< 0.001
	Item 11	0.651	0.0605	0.532	0.769	10.75	< 0.001
	Item 12	0.588	0.0525	0.485	0.691	11.20	< 0.001
	Item 13	0.569	0.0571	0.457	0.681	9.96	< 0.001
	Item 14	0.551	0.0477	0.457	0.644	11.56	< 0.001
	Item 15	0.571	0.0541	0.465	0.677	10.56	< 0.001

The study employed a sample of 153 registered nurses who voluntarily completed the questionnaire. This sample represented the Spanish nursing population, with characteristics closely mirroring the national demographics: 86.9% were female, 61.4% held a master's degree, 32.7% were between 50 and 59 years old. The sample's representativeness was confirmed by comparison with the Spanish National Institute of Statistics (INE) data from 2021 (National Statistics Institute n.d.), which showed: 84.2% of nurses are women, and 35.3% are between 45 and 65 years old.

This single sample of 153 nurses was used across the psychometric stages of exploratory factor analysis (EFA) and Confirmatory factor analysis (CFA). While using the same sample for both EFA and CFA is not ideal from a methodological perspective, it is sometimes necessary in smaller studies due to practical constraints (Kyriazos 2018). The robust statistical findings, including a Kaiser‐Meyer‐Olkin (KMO) measure of 0.922 and significant Bartlett's Test of Sphericity, provide compelling evidence for the questionnaire's construct validity despite this limitation.

- *Bartlett's Test of Sphericity* yielded significant results (*χ* ^2^ = 1498, df = 105, *p* < 0.001), confirming sufficient correlation among variables for factor analysis (Taber 2018).
- *Kaiser‐Meyer‐Olkin (KMO)* measure of sampling adequacy was calculated with a global value of 0.922, considered excellent. Individual item MSA values ranged from 0.878 to 0.950, all above the recommended threshold of 0.8 (Taber 2018).

### 3.5 Model Fit Indices

Model fit indices from confirmatory factor analysis suggested potential improvements in model fit to data: RMSEA value was found to be 0.106 (90% CI: 0.0894–0.124) TLI was reported at 0.867. These results indicate that while the questionnaire demonstrates good construct validity regarding its underlying structure, a larger sample may be necessary for achieving a better fit with observed data.

### 3.6 Result of Clinical Reasoning Descriptive Study

The mean score and the standard deviation of the items were calculated. The mean score shown in Table 5 ranged between 3.76 and 4.37, and the standard deviation ranged between 0.68 and 0.92.

**TABLE 5:** Mean score and standard deviation for NCRS items (*N* = 153).

Items NCRS Spanish version	Mean	(SD)
1. I know how to collect an admitted patient's health information quickly	4.16	(0.92)
2. I can apply proper assessment skills to collect a patient's current health information	4.17	(0.76)
3. I can identify abnormalities from the collected patient information	4.19	(0.68)
4. I can identify a patient's health problems from the abnormal information collected	3.89	(0.92)
5. I can recognise possible early signs or symptoms when a patient's health deteriorates	4.26	(0.73)
6. I can explain the mechanism and development associated with the early signs or symptoms when a patient's health deteriorates	3.90	(0.80)
7. I can accurately prioritise and manage any identifiable patient problems	3.89	(0.84)
8. I can correctly explain the mechanism behind a patient's problems	3.76	(0.82)
9. I can set nursing goals properly for the identified patient problems	4.06	(0.73)
10. I can provide appropriate nursing intervention for the identified patient problems	4.14	(0.70)
11. I am knowledgeable of each nursing intervention provided	4.10	(0.87)
12. I can identify and communicate vital information clearly to the doctors based on the patient's current condition	4.30	(0.76)
13. I can anticipate the prescription ordered by the doctor according to the patient information provided	4.00	(0.80)
14. I can accurately evaluate and identify whether a patient's condition is improved	4.37	(0.70)
15. I know the follow‐up steps to take if the patient's condition does not improve	4.12	(0.77)

The level of clinical reasoning of the groups, described in Table 6, was measured with the NCRS to analyse the relationship between the demographic variables and the clinical reasoning: age group, gender, academic degree and work ward.

**TABLE 6:** Level of clinical reasoning by groups.

Variable	CR mean	*p*
*Age group*		
20–29	60.10	< 0.001
30–39	61.17	< 0.001
40–49	60.70	< 0.001
50–59	62.62	< 0.001
60–65	61	< 0.001
*Gender*		
Female	62.06	< 0.001
Male	57.05	< 0.001
No gender	—	
Not say	—	
*Academic degree*		
Only nursing degree	59.30	< 0.001
Post‐graduate	59.42	< 0.001
Master	61.68	< 0.001
PhD	66.16	< 0.001
*Ward*		
Medical and surgical ward	61.95	< 0.001
Others (psychiatry, haemodialysis operating room)	61.81	< 0.001
Obstetrics or paediatrics	62.91	< 0.001
Emergencies or intensive care	58.53	< 0.001

The clinical reasoning level in the group between 50 and 59 years has the highest level, with a mean of 62.62 points on the scale, and in the group between 20 and 29 years had the lowest, with a mean of 60.10 points on the scale. In reference to the academic degree, the nurses who only had a nursing degree obtained the lowest punctuation with 50.30 points, and the nurses who had a PhD had the highest punctuation, 66.16. In relation to the workplace, the nurses who work in the obstetrics or paediatrics wards obtained the highest level of clinical reasoning with a mean result of 62.91. The general level of clinical reasoning of the nurses in this study was a mean of 60.98 CR.

## 4 Discussion

The aim of this study was to translate the Nurse Clinical Reasoning Scale (NCRS) into Spanish, to examine its psychometric properties, and to know the Spanish nurses’ clinical reasoning level. This study tested and described for the first time the translation of the NCRS to the Spanish language.

The questionnaire was completed voluntarily by 153 registered nurses. The largest group was females, 86.9% from 50 to 59 years of age, and 61.4% of the sample had a master's degree. The sample had a good clinical reasoning level with 60.98 mean score of 60.98 points out of the 75 maximum points on the scale. According to the Spanish National Institute of Statistics (INE), in 2021, there were 330,745 registered nurses in Spain, of which 84.2% were women, and 35.3% were between 45 and 65 years old (Antolí‐Jover et al. 2024). The sample of our study represents the study population that is 87% women and 36% between 50 and 65 years old. Therefore, our sample represents, in age and gender, the Spanish nursing population.

The questionnaire's construct validity has been thoroughly validated using both exploratory and confirmatory factor analyses. The Bartlett's Test of Sphericity produced significant results, and CVI was 0.93, confirming a good validity of the scale, demonstrating that the variables are adequately interrelated for factor analysis. Moreover, the Kaiser‐Meyer‐Olkin (KMO) measure yielded a global value of 0.922, which is considered excellent in statistical terms (Taber 2018). This high KMO value not only confirms the data's suitability for factor analysis but also indicates that each item in the questionnaire makes a substantial contribution to the overall factorial structure. These robust statistical findings collectively provide compelling evidence for the questionnaire's construct validity and the soundness of its structural composition. The Cronbach alpha for the original scale was 0.93, indicating that the adaptation of the scale to the Spanish language maintained a good internal consistency. The Spanish version of the Nursing Clinical Reasoning Scale (NCRS) demonstrated robust psychometric properties, aligning with other international versions. Recently, the NCRS has been translated to Farsi language (Kojabadi et al. 2023), both the Persian and Spanish versions of the NCRS exhibited strong internal consistency, with Cronbach's alpha values of 0.9 and 0.93, respectively, and the content validity was excellent in both versions. This similarity suggests that the NCRS maintains its reliability across different cultural and linguistic contexts, supporting its use as a consistent measure of clinical reasoning in diverse nursing populations.

The Spanish translation is now available and could be applied to 500 million people in the world. Spanish is the second mother tongue in the world (El español en el mundo 2014); therefore, this translation version opens a new opportunity to study nurses' CR in Spanish‐speaking countries.

Besides, we analysed the relation between clinical reasoning level and the socio‐demographic characteristics of the participants. The nurses who participated in this study had a high level of training (four years of university education with 360 ECTS), with the majority having a master's degree. Sermeus (Aiken et al. 2014) identified that higher nurses' education correlates with better patient outcomes, concluding that hospitals with bachelor's degrees nurses had significantly lower mortality rates compared to hospitals with fewer bachelor's degrees among nurses. Moreover, there is an important relationship between higher nurses ‘education and patient safety (Aiken et al. 2014).

The study was conducted in some of Spain's most renowned and prestigious hospitals, where nurses have vast experience and high level of post‐graduate education such as master's degrees, then in consequences these hospitals may have better working conditions or environments, so their results cannot be extrapolated to other healthcare centres. Nurses in the studied hospitals work based on protocols and good practice guidelines and they are also in charge of educating nursing students in their clinical practice (Rodríguez Serrano 2018). These results showed that there is no significant difference in the clinical reasoning between nurses in ICUs and medical‐surgical wards, which is consistent with the findings of other studies (Babamohamadi et al. 2017).

The results of the NCRS assessment for Spanish nurses highlighted certain areas of needed improvement. The lowest average response was recorded for item eight, which pertains to the correct explanation of the mechanism behind a patient's problems, with a mean response of 3.76 over five. This was followed by items four and seven, which relate to identifying a patient's health problems from collected abnormal information and accurately prioritising and managing any identifiable patient problems, respectively. The mean average score for these items was 3.89. The areas of improvement presented in this study may be related to the fact that 32.7% of the nurses belonged to the 50–59‐year‐old group and some nurses rely on habits rather than clinical reasoning during their shifts, only utilising these skills in unfamiliar situations (Ali‐Abadi et al. 2020), besides Spanish nurses rated worst in item four ‘Identifying a patient's health problems from collected abnormal information’ probably because in the Spanish context, nurses do not perform patient assessment thorough a comprehensive physical examination, because medical staff do it within the team work activities. In Spain, the ratio of physician is 4.5 practising physician per 1000 population over the OECD average of 3.7 (OECD 2023), and they performed the physical examination during the patient hospitalisation. Therefore, nurses may probably lose some relevant information by not performing this comprehensive physical assessment. The results obtained in item seven, ‘I can accurately prioritize and manage any identifiable patient problems’, and item eight ‘I can correctly explain the mechanism behind a patient's problems’ could be explained because, unlike the average of the physicians, Spain has below the European average of nurses for every 1000 inhabitants, with 6.3 practising nurses (OECD average 9.2) (OECD 2023) this is one reason why nurses do not have enough time to do their professional activities using all their knowledge obtained during the nursing degree. In Spain, all nurses are trained with a four‐year university bachelor's degree that meets the European standards of healthcare (Cabrera and Zabalegui 2020), ensuring top‐tier medical assistance to patients. However, their job responsibilities often come with a significant workload that could affect the CR process. The heavy workload may hinder quick decision‐making, for instance they do not have enough time for systematically debriding group conversation potentially resulting in unfavourable patient safety and care outcomes (Holder 2018).

On the other side, the item with the best average response was recorded for item fourteen, which pertains to evaluating and identifying whether a patient's condition is improved, with a mean average of 4.37. Besides the following item is the twelve, which is defined by identifying and communicating vital information clearly to the doctors based on the patient's current condition, with a mean average of 4.30. According to 2023 OECD data, Spain performs better than the OECD average on 54% in quality care indicators (OECD 2023).

A comprehensive understanding of the levels of nurse's clinical reasoning in our context can help us identify areas for improvement and achieve better outcomes for patients. By enhancing our clinical reasoning abilities, we can provide a higher standard of care and safety overall.

The application of clinical reasoning in nursing practice is a critical component of delivering high‐quality patient care. The relationship between clinical reasoning skills and patient outcomes represents a fundamental aspect of high‐quality healthcare delivery (Liou et al. 2016); clinical reasoning enables nurses to analyse complex patient situations, make informed decisions and implement appropriate interventions (Benner et al. 2008). This structured approach allows nurses to navigate the dynamic and often unpredictable nature of healthcare settings, ensuring patient safety and improving overall outcomes. By employing clinical reasoning skills, nurses can identify potential complications early, prioritise care and make critical decisions that directly impact patient recovery and satisfaction (Borzo et al. 2024). Furthermore, the application of clinical reasoning in nursing practice extends beyond individual patient care to influence the broader healthcare system. Nurses with strong clinical reasoning abilities contribute to improved treatment outcomes, increased patient satisfaction and more efficient use of healthcare resources (Levett‐Jones et al. 2010). As the healthcare landscape becomes increasingly complex, the emphasis on developing and refining clinical reasoning skills in nursing practice remains paramount for ensuring high quality.

The findings of this study have significant implications for both nursing education and clinical practice. In nursing education, the validated Spanish version of the NCRS can be used as a tool to assess and develop clinical reasoning skills among nursing students and practicing nurses. Educators can design targeted interventions to address the areas of improvement identified, such as explaining patient problem mechanisms and prioritising patient issues. In clinical practice, healthcare institutions can utilise the NCRS to evaluate and enhance nurses' clinical reasoning abilities, potentially improving patient outcomes and safety. The scale can also be incorporated into continuing education programmes and performance evaluations. By focusing on these areas, nursing education programmes and healthcare facilities can work towards cultivating a workforce with strong clinical reasoning skills, ultimately contributing to higher quality patient care and improved healthcare outcomes in Spanish‐speaking contexts.

## 5 Limitations

It is necessary to take into account that The Nursing Clinical Reasoning Scale (NCRS) relies on self‐perception, which can introduce bias as individuals may have inaccurate self‐assessments. This self‐perception aspect can influence the results and should be considered when interpreting the findings. Also, it is necessary to consider that the study sample was not randomised and included nurses from high‐quality ranking hospitals, with a significant portion of participants aged 50 to 59, indicating a high level of experience. These factors introduce several limitations. The lack of randomisation means the sample may not be representative of the broader nursing population. This limits the ability to generalise the findings to other settings. Focusing on high‐quality hospitals may mean that the findings are not applicable to lower‐ranked hospitals, which may have different challenges and levels of resources. The predominance of highly experienced nurses (aged 50 to 59) could skew the results, as their perceptions and competencies might differ significantly from those of less experienced nurses. Future studies should include nurses from different age groups, levels of experience and types of hospitals (e.g., lower‐ranked, rural and community hospitals) to enhance the generalisability of the findings, and implementing randomised sampling methods would help ensure that the study sample is representative of the broader nursing population, thereby improving the validity of the results. Conduct thorough cultural adaptation processes, including translation and back translation, pilot testing in diverse regions and engaging local experts to refine the tool for different cultural contexts. The results of this study are conditioned by the characteristics of the sample and the specific hospitals that participated. Acknowledging these limitations and addressing them in future research will enhance the robustness and applicability of the findings across different nursing populations and hospital settings.

The Spanish language and cultural context vary significantly across regions, such as Spain and Latin America. Therefore, a cultural adaptation of the tool is necessary to ensure its relevance and accuracy in different contexts. Spanish dialects vary widely. Words, phrases and idiomatic expressions can have different meanings in different countries. Ensuring that the tool uses language that is universally understood or appropriately localised is crucial. Besides, cultural attitudes towards behaviour's, health practices and social norms can differ. A tool developed in one cultural context might not resonate or be as effective in another without adjustments. The context in which behaviour's occur can vary.

On the other hand, we did not conduct the confirmatory factor analysis because it required from 300 to 500 subjects (Kyriazos 2018). Therefore, the results of the study can be conditioned to the characteristics of the sample and the hospitals that were willing for participate in this study. In futures research to strengthen the validity of the tool, conduct confirmatory factor analysis (CFA) with an adequate sample size (300 to 500 subjects) to ensure the tool's structure is sound.

## 6 Conclusions

The NCRS is a valid and reliable instrument, and the Spanish version can be used to evaluate the clinical reasoning level of nurses in the Spanish context. The study shows that Spanish nurses who participated in the study have a good level of CR, especially in those with more education, like a master's degree. We can affirm that clinical reasoning ability is present in nurses with higher education level. In general, Spanish nurses' who participated in the study are equipped with the necessary skills and knowledge to provide the best possible care and safety to their patients, having a clear understanding of the patient's condition, implementing the right interventions at the right time, which can lead to better patient outcomes. The study identified areas for potential improvement in clinical reasoning among the participants, specifically in physical assessment techniques and patient problem prioritisation skills.

## Author Contributions

A.P.P., N.F. and A.Z. made substantial contributions to conception and design, or acquisition of data, or analysis and interpretation of data and agreed to be accountable for all aspects of the work in ensuring that questions related to the accuracy or integrity of any part of the work are appropriately investigated and resolved. A.P.P. and A.Z. were involved in drafting the manuscript or revising it critically for important intellectual content. A.Z. given final approval of the version to be published. Each author should have participated sufficiently in the work to take public responsibility for appropriate portions of the content.

## Ethics Statement

The study has the approval of the Ethics and Research Committee of the Hospital Clinic of Barcelona (register number: HCB/2022/0955).

## Conflicts of Interest

The authors declare no conflicts of interest.

## Data Availability

Research data are not shared.
